# Early Desquamating Perineal Erythema in a Febrile Infant: A Characteristic Clinical Feature of Kawasaki Disease

**DOI:** 10.3390/ijerph14070710

**Published:** 2017-06-30

**Authors:** Chiara Isidori, Lisa Sebastiani, Maria Chiara Cardellini, Giuseppe Di Cara, Donato Rigante, Susanna Esposito

**Affiliations:** 1 Pediatric Clinic, Department of Surgical and Biomedical Sciences, Università degli Studi di Perugia, 06132 Perugia, Italy; chiaraisi88@gmail.com (C.I.); sebastianilisa@tiscali.it (L.S.); mchiara.cardellini@gmail.com (M.C.C.); giuseppe.dicara@unipg.it (G.D.C.); 2 Institute of Pediatrics, Università Cattolica Sacro Cuore, Fondazione Policlinico Universitario A. Gemelli, 00168 Rome, Italy; drigante@gmail.com

**Keywords:** atypical Kawasaki disease, coronary artery abnormalities, echocardiography, erythematous perineal rash, Kawasaki disease

## Abstract

*Background*: The occurrence of a distinctive perineal eruption that appears early in infants with Kawasaki disease (KD), the most relevant type of febrile vasculitis of childhood, has received little attention in pediatric reports. KD diagnosis is based on clinical criteria, which can be supported by laboratory abnormalities or positive echocardiography findings: difficulty in diagnosis can be increased by incomplete or atypical presentations, but a timely diagnostic process is essential in the youngest patients who are more prone to the risk of cardiac sequelae resulting from KD. *Case Presentation*: In this report, we present the case of a 2-month-old infant with an unusual presentation of KD, in whom diagnosis was made despite fever remission on the fourth day of hospitalization following intravenous corticosteroid therapy to treat concomitant bronchoconstriction. The presence of early desquamating perineal erythema led to the consideration of KD diagnosis, confirmed by the echocardiographic assessment of right and left coronary artery dilatations with pericardial effusion on the fifth day of hospital stay. *Conclusions*: Diagnosis of KD represents a demanding challenge, mainly when the illness starts with an incomplete or nuanced presentation. An erythematous desquamating perineal rash is a valuable early clinical clue, which might facilitate a prompt diagnosis of KD. This case emphasizes that an accurate assessment of all clinical features, including perineal erythema with early tendency to desquamation, and an eventual echocardiography, are necessary in an infant displaying fever to corroborate the suspicion of KD.

## 1. Background

Kawasaki disease (KD) or “mucocutaneous lymph node syndrome” is an acute, immune-mediated, self-limiting vasculitis of childhood, which is triggered by the release of substances after exposure to unknown agents. It mainly affects children under 5 years of age, and can result in coronary artery abnormalities (CAA) in previously healthy children. It is the most common cause of acquired heart disease in children living in the developed world [1,2]. The estimated overall annual incidence of KD in Europe is 5–10 per 100,000 children, about 80% of whom are younger than 5 years of age, with the peak age incidence from 6 months to 2 years [3]. As in other childhood immune-mediated diseases, age of around 5 years seems to be the turning point for immune maturation of the host and KD may be associated with maturing immune system of young children. In Asian countries (i.e., Japan, South Korea, Taiwan, and China) a period of economic growth, industrialization and westernization has been associated with the appearance of KD [1,2,3]. The systemic vascular inflammation involves small and medium-sized arteries, and has a peculiar predilection for the coronary arteries [4].

Classically, diagnosis of KD is based on clinical criteria represented by persistent fever for five or more days with at least four of five main clinical features, which tend to appear sequentially: bilateral nonexudative conjunctivitis, erythema of the lips and oral mucosa, polymorphous rash, cervical lymphadenopathy, and abnormalities of hands and feet, including also perineal erythema (Table 1) [5]. Typically, all these clinical features are not present at the time of fever onset. Furthermore, KD occasionally manifests itself in incomplete or atypical presentations. Incomplete KD is more common in infants younger than 12 months or older than 5 years and is characterized by unexplained fever for more than five days, variably associated with two or three characteristic clinical features. Additionally, diagnosis of KD should be considered in any infant under 6 months with fever for at least seven days, laboratory evidence of systemic inflammation, and echocardiographic imaging of CAA, with no other explanation of the febrile illness [6].

Approximately, 15–25% of untreated KD patients may develop CAA, including aneurysms or ectasia; this condition may lead to myocardial infarction, ischemic heart disease, or even sudden death [7,8]. Prompt treatment of KD with intravenous immunoglobulin (IVIG) and acetylsalicylic acid (ASA) within ten days of disease onset reduces the prevalence of CAA to 2–4%. An accurate evaluation of all presumed KD typical signs and symptoms appearing sequentially, usually not simultaneously during the physical examination, is mandatory, and perineal erythema with early desquamation should be considered pathognomonic of KD [9]. The timely diagnosis and treatment are essential in these young patients who are at a substantial risk of developing CAA [10]. We herein present the case of an infant for whom diagnosis of KD was suggested due to early-desquamating perineal erythema despite fever remission on the fourth day of hospital stay.

## 2. Case Presentation

A 2-month-old male infant was admitted to our hospital due to a brief, resolved unexplained event: his personal history showed full-term birth, perinatal well-being, and normal motor development. On the first day of hospitalization, fever appeared, associated with wheezing and clinical signs suggestive of bronchiolitis. Nasal swab resulted positive for respiratory syncytial virus. Chest X-ray film showed mild interstitial lung disease. On the second day of hospitalization, cracked lips and perineal erythema appeared. At that time laboratory studies showed an elevation of white blood cells (WBC, 13,500/mm^3^, with 70% of neutrophils), high C-reactive protein (CRP, 8.8 mg/dL, normal value <0.5), and high erythrocyte sedimentation rate (32 mm/1 h), while other blood parameters were within normal ranges (platelet count 411,000/mm^3^, hemoglobin 10 g/dL). Blood culture, urinalysis, and serology against Epstein–Barr virus, cytomegalovirus, adenovirus, and parvovirus were all negative.

Due to the increase in WBC, neutrophils and CRP, in the hypothesis of a viral and bacterial co-infection, empirical antibiotic therapy was started with ampicillin/sulbactam. Intravenous corticosteroid therapy with methylprednisolone (1 mg/kg/day) was also added on the third day of hospitalization, due to respiratory deterioration and bronchoconstriction. Furthermore, a macular rash appeared on the trunk, with worsening of the perineal erythema and tendency to desquamation.

On the fourth day of hospitalization, fever disappeared. The suspicion of KD was placed in the presence of three characteristic clinical features (extensive cutaneous rash, fissuring and cracking of the lips, and perineal erythema), although there was no fever. Additional criteria to establish diagnosis of KD were evaluated: laboratory studies showed increased CRP (7 mg/dL) and platelet count (470,000/mm^3^) with low hemoglobin (8.7 g/dL). On the other hand, serum transaminases, serum gamma glutamyl-transpeptidase, sodium, albumin, total bilirubin, fibrinogen, and serum immunoglobulins were all normal. Urinalysis for pyuria, abdominal ultrasound for hydrops of the gallbladder, and eye examination were performed, but were unrevealing.

**Figure 1:**
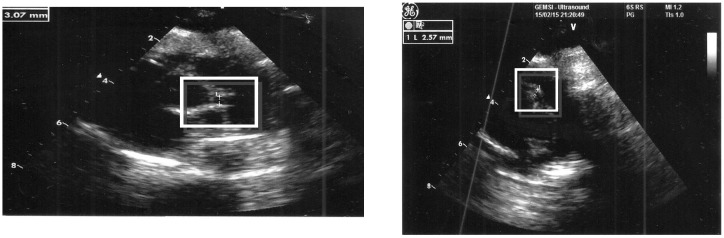
Slight right and left coronary dilatation: 3 mm for the right, 2.57 mm for the left.

On the fifth day of hospitalization, echocardiography showed the presence of slight coronary artery dilations (right coronary artery diameter: 2.57 mm, left coronary artery: 3 mm) and pericardial effusion (Figure 1). Electrocardiography and troponin were normal.

Diagnosis of incomplete KD was then made. A single dose of IVIG (2 g/kg of body weight) was administered in combination with daily ASA which was given at the anti-inflammatory dosage of 60 mg/kg/day, continued until reduction of inflammatory markers, and then switched to the anti-platelet dose of 3 mg/kg/day. Desquamation of the periungueal region of hands extending to the whole of the fingers appeared on the tenth day of hospitalization, when platelet count was further increased (655,000/mm^3^). Inflammatory markers were normalized at the fourteenth day of hospitalization (CRP 0.8 mg/dL), while coronary artery diameters remain unchanged.

The patient was then discharged with ASA-based anti-platelet therapy and put into cardiologic follow-up. The first cardiac ultrasound, four weeks after disease onset revealed an increased diameter of coronary arteries (right coronary artery: 4 mm, left coronary artery: 5 mm) in the absence of pericardial effusion. Platelet count was 496,000/mm^3^. Eighteen months later echocardiography showed a slight residual dilation of the left coronary artery, equal to 2 mm, and a complete regression of the right coronary artery abnormality. At that time, laboratory tests were normal (CRP 0.1 mg/dL, platelet count 210,000/mm^3^, WBC 6700/mm^3^, with 27% of neutrophils, hemoglobin 12.5 g/dL). Currently, the infant receives ASA prophylaxis, which will be maintained until echocardiographic resolution of the left coronary artery abnormality.

This case report was approved by the Ethics Committee of Azienda Ospedaliera di Perugia, Perugia, Italy. For the case reports, Azienda Ospedaliera di Perugia, Perugia, Italy, does not provide a reference number. Written informed consent for the publication of these case reports and any accompanying images were obtained from the patients’ parents.

## 3. Discussion

Endothelial cell and cardiomyocyte injury, neutrophil and platelet aggregation, and immune overactivation which amplifies reciprocal vascular inflammatory reactions, are hallmarks of acute KD and cardiac KD complications [11]. Many studies have tried to define which clues can predict the occurrence of CAA: higher values of CRP and younger age at onset are crucial in determining, respectively, a failure in the response to IVIG and an increased occurrence of CAA [12].

In our case fever onset was associated with a respiratory airway disease, caused by respiratory syncytial virus, and specific supportive measures to control this infection probably masked the natural course of KD. Diagnosis of a viral or bacterial infection cannot exclude KD, considering the multifactorial etiology and the presumed infectious environmental triggers of the disease [13]. In fact, various data suggest that KD is intimately related to an infectious agent and that its clinical expression is influenced by a predisposing genetic background. Other epidemiological studies have also supposed the role of noninfectious causes such as allergens, immunizations and normal flora variants caused by environmental changes (such as improved public health hygiene or a western lifestyle in Asian countries) in the pathogenesis of KD [14].

The skin rash of KD is usually truncal, with protean characteristics changing from macular and papular or urticarial to morbilliform and scarletiniform: it can be sometimes localized in the diaper area, and differential diagnosis for an erythematous desquamating rash in the perineal areas should include bacterial, viral, and yeast infections [15,16,17]. Another condition to differentiate is recurrent toxin-mediated perineal erythema, a skin eruption characterized by erythematous macular or pustular lesions involving the perineum, caused by superantigen toxins produced by various streptococci, which quickly improves after antibiotic treatment [18].

In one large series of KD patients, Aballi reported that the KD rash might appear in the diaper area in 62.5% of cases observed: the perineal eruption was described as a confluent, sometimes tender, macular to plaque-type erythema involving part or all of the perineal region, which can be shortly followed by desquamation [19]. Sparing of the groin folds, prominent maceration, vesiculation, and pustulation are not part of the KD clinical picture. Perineal erythema and desquamation appeared more frequently in a series of children with KD evaluated at the Children’s Hospital Medical Center, in Cincinnati, than typical KD oropharyngeal changes, making this finding as highly contributive to the diagnosis of KD [20]. When the eruption is found in conjunction with any of the other classically described KD criteria, it may provide a clue early in the course of the illness for further investigation. Cardiac evaluation and potentially life-saving therapy can then be embarked on days before other mucocutaneous findings appear. Therefore, the recognition of this cutaneous finding as a part of KD may prevent physicians from making erroneous diagnoses and delaying the diagnostic process of KD, which may prevent substantial cardiac morbidity [20].

It is believed that normal flora (or microbiota) and the host have a reciprocal benefit relationship and normal flora may not induce severe inflammation when they invade into the host. After invasion of KD agents, the patients may have a focus where the inflammatory mediators, including replicated KD agents, byproducts of KD agent replication process, and the substances from activated immune cells and injured host cells, are produced [21,22]. In this stage, majority of the KD agent-infected patients may be asymptomatic, with a self-limiting reaction. However, during this process, KD develops when the substances from a focus spread into systemic circulation. Various clinical signs of multiple organ involvement (including clinical diagnostic criteria) in KD may originate from these systemically spread substances (i.e., trigger factors) and corresponding immune cells. Since the probability of invasion of KD agents into the host in young children may be similar among the populations, the ethnic different incidence of KD could be explained by the fact that KD patients in Asian countries and Hawaii may have more chances for exposure to and colonization of KD agents, and the distribution of microbiota in parents/guardians of young children in these regions may differ to Western countries and developing countries [23]. The host immune system, including macrophage-linage cells, controls not only the pathogen-derived substances such as viral RNAs and DNAs (PAMPs) but also the substances known as damage (danger)-associated molecular patterns (DAMPs) that may be derived from host cells injured by infectious insults [21,22]. This control system (the protein-homeostasis-system) of the host is important for recovery from KD. Therefore, severely affected KD patients with giant aneurysms may have an improper immune/repair system against the substances from KD agents or injured coronary artery cells.

In our patient, despite apyrexia on the fifth day of illness, diagnosis of KD was re-considered because of the echocardiographic evidence of CAA associated with three suggestive clinical features, among which early-desquamating perineal erythema resulted the most frankly evocative of KD. Almost all KD patients that have rapid defervescence after IVIG treatment show no CAA progression within a month, although rarely some patients progress to severe CAAs, including giant aneurysms, after early defervescence [19,24]. This finding suggests that the severity and chronicity of CAAs may depend on the host’s immune status against the insults from KD. Further additional laboratory features, insufficient to substantiate KD diagnosis, were also considered in our patient: low hemoglobin and thrombocytosis together with increased CRP. Therefore, six days after disease onset, the appropriate treatment with IVIG and ASA was started. The zenith of the platelet count was reached on the tenth day of disease, when the characteristic periungueal skin desquamation of fingers in both hands also appeared.

Indeed, the use of intravenous corticosteroid treatment on the third day of hospital stay to control the child’s bronchoconstriction probably led to fever remission within 24 h, though its dosage was inadequate to manage KD. After sixteen months, the last echocardiographic evaluation continues to show minimal left coronary artery involvement, requiring the patient to continue anti-platelet prophylaxis (until the dilation is resolved). Many recent studies have supported the association of corticosteroids with IVIG in the initial management of children with KD, mostly to enhance the efficacy of IVIG and reduce the overall risk of CAA [19]. The prediction of IVIG resistance is a matter of debate, as identified high-risk patients should consent the administration of intensified initial treatment in combination with IVIG. Few reports have focused on factors, referring to both clinical parameters and laboratory data at the onset of KD, in order to predict which patients might be IVIG non-responsive [25].

## 4. Conclusions

Diagnosis of KD represents a demanding challenge, mainly when the illness starts with an incomplete or nuanced presentation. An erythematous desquamating perineal rash is a valuable early clinical clue which might facilitate a more prompt diagnosis of KD. We emphasize that an accurate assessment of all focused clinical features, including perineal erythema with early tendency to desquamation, and an eventual echocardiography, visualizing the potential occurrence of CAA, are necessary in an infant displaying fever to corroborate the suspicion of KD, start proper treatment early, and decrease the risk of cardiac sequelae.

## Figures and Tables

**Table 1:** Clinical criteria for diagnosis of Kawasaki disease in the acute phase: fever is a mandatory criterion associated with at least four of five classic clinical signs. Additional laboratory data may be helpful in the diagnostic process of Kawasaki disease, but they have not been validated yet.

Fever Persisting for at Least 5 Days
Presence of at Least Four Principal Features:
1. Bilateral nonexudative conjuntivitis
2. Mucositis: cracked red lips and/or strawberry tongue
3. Skin rash: polymorphous exanthema
4. Changes in the extremities: erythema of the palms and/or soles and edema of hands and/or feet
5. Cervical lymph node enlargement (>1.5 cm), usually unilateral
Additional Laboratory Data:
1. Serum albumin ≤3.0 g/dL
2. Anemia with respect to age
3. Elevation of alanine aminotransferase
4. Platelets after 7 days of disease ≥450,000/mm^3^
5. White blood cells (WBC) ≥15,000/mm^3^
6. Sterile pyuria
7. C reactive protein ≥3 mg/dL
8. Erythrocyte sedimentation rate ≥40 mm/h
